# Lumbar Radiculopathy Caused by Epidural Gas Collection

**DOI:** 10.1155/2022/8338131

**Published:** 2022-05-30

**Authors:** Dong Hu, Kai Xu, Songhua Xiao

**Affiliations:** Department of Orthopedic, Beijing Tsinghua Changgung Hospital, School of Medicine, Tsinghua University, Beijing, China

## Abstract

**Background:**

Degenerated intervertebral discs in the lumbar spine are commonly found with vacuum phenomenon. In a few cases, gas can migrate into the lumbar spinal canal and compress the nerve root. *Case Presentation*. We report a case of lumbar radiculopathy caused by epidural gas collection in a 59-year-old woman. Originally, the gas was formed in the intervertebral disc and possibly migrated backward because of the motion of lumbar spine, forming a single large gas formation. The nerve root was freed from the gas-filled cyst after needle puncture was performed. Patient's symptoms in the leg were significantly relieved following surgery.

**Conclusion:**

There is still no satisfactory explanation for the pathogenesis of gas formation in the spinal canal. In our case, the presence of gas in the spinal canal and gas inside a narrowed disc suggests a communication between the two structures.

## 1. Introduction

It is rare for intraspinal gas to cause lumbar radiculopathy, and there are only a few relevant case reports [1–6]. It is possible that epidural gas may be misdiagnosed as epidural masses or herniated intervertebral discs by magnetic resonance imaging (MRI) [7]. Computed tomographic (CT) scan is the preferred investigation to diagnose it as seen in all cases in literature. We recently encountered a case of lumbosacral radiculopathy caused by epidural gas collection in the lumbar region.

## 2. Case Presentation

**Figure 1:**
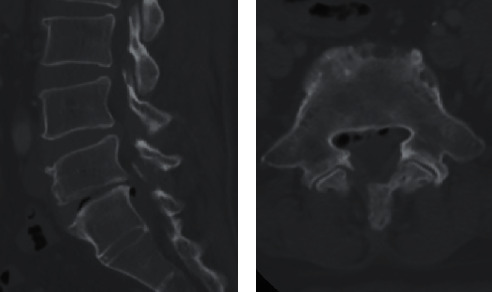
CT scan images revealing multiple gas bubbles at L5/S1 level as shown in axial (a) and sagittal (b) cuts.

A 59-year-old woman presented with right leg pain that first appeared in January 2019. Axial CT revealed multiple intracannular posterolateral gas bubbles at L5-S1 level (Figure 1). A coexisting vacuum disc phenomenon indicates the presumed origin of the gas bubbles. There was no history of invasive procedures such as epidural injection or surgery. Conservative treatment had been successful. Partial relief of pain had been achieved during the following two years.

**Figure 2:**
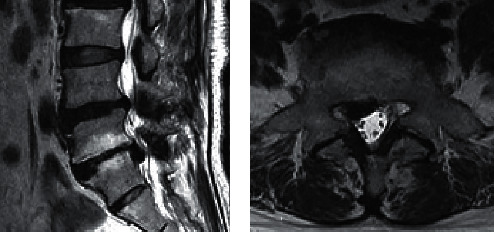
T2 sequences in sagittal (a) and axial orientation (b) illustrate a caudally shifted hypointense mass in the right lateral recessus L5/S1 with contact to the nerve root S1 suspicious of a sequestered disc herniation.

**Figure 3:**
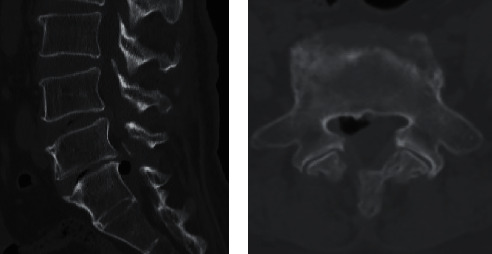
CT reveals a single large epidural gas bubble mimicking disc herniation on MRI as shown in axial (a) and sagittal (b) cuts. A coexisting vacuum disc phenomenon indicates the presumed origin of the gas bubble.

Since March 2021, however, patient's ability to walk gradually deteriorated due to recurrence of the symptom. Her symptom had not improved with conservative therapy. A positive straight leg raising test at 45° was found in right leg without any motor weakness on physical examination. According to MRI results, a well-encapsulated low signal intensity lesion was related to gas formation or calcification (Figure 2). An additional CT was performed, revealing a large intraspinal epidural bubble compressing the right S1 nerve root (Figure 3). An ipsilateral percutaneous L5/S1 interlaminar approach endoscopic surgery was performed with enlargement of the lateral recess. At surgery, the S1 nerve root appeared swollen and the gas-containing pseudocyst was observed at the axilla region of the nerve root, covered with a thin capsule. There was no herniated disc around the pseudocyst. The nerve root was freed from the gas-filled cyst after needle puncture was performed. Patient's symptoms in the leg were significantly relieved following surgery. The patient was pain-free 6 months after the surgery.

## 3. Discussion

Epidural gas is mainly composed of nitrogen and is frequently trapped within a thin layer of nonspecific fibrous tissue membrane that is similar to the posterior longitudinal ligament [8, 9]. Clinical symptoms of epidural gas can be similar to those of other more common causes of lumbar radiculopathy [10]. There have been very few reports in the literature of symptomatic lumbar radiculopathy caused by a gas bubble.

There is still no satisfactory explanation for the pathogenesis of gas formation in the spinal canal. In our case, the presence of gas in the spinal canal and gas inside a narrowed disc suggests a communication between the two structures. Possibly, the motion of the lumbar spine causes a pressure gradient, resulting in a valve-pump mechanism that propels the gas into the spinal canal. Gas in the epidural space usually disappears spontaneously in most patients, so conservative treatment and a period of observation are recommended before surgery [11]. In our case, the patient had multiple gas bubbles at L5/S1 level. The bubbles were not absorbed spontaneously but forming a single large gas formation which leads to surgery two years later. Decompression and fusion surgery could be used in patients with spine instability and spinal canal stenosis, whereas simple microscopic/endoscopic removal of cyst could be sufficient in patients without spine instability and spinal canal stenosis. In this case, we offered different operation options for her to choose from, including fusion surgery, endoscopic surgery, and CT-guided needle puncture. The patient picked endoscopic surgery in consideration of lower cost and less invasive procedure compared with fusion surgery, while lower recurrence rate compared with CT-guided needle puncture. The patient was pain-free 6 months after the surgery, while a longer follow-up period is required to fully observe the long-term recurrence of the cyst lesion in this patient.

## 4. Conclusion

Intraspinal gas collection is a rare cause of lumbar radiculopathy. We speculate that the motion of the lumbar spine induces a pressure gradient, resulting in a valve-pump mechanism that propels the gas into the spinal canal. Surgery should be considered when conservative therapy failed.

## Data Availability

The patient's data used in this study are restricted by the Ethics Board of Tsinghua Changgung Hospital in order to protect patient privacy.
